# Association Between Hearing Characteristics/Prognosis and Vestibular Function in Sudden Sensorineural Hearing Loss With Vertigo

**DOI:** 10.3389/fneur.2020.579757

**Published:** 2020-12-17

**Authors:** Yixu Wang, Le Wang, Yuanyuan Jing, Lisheng Yu, Fanglei Ye

**Affiliations:** ^1^Department of Otorhinolaryngology, Head and Neck Surgery, People's Hospital, Peking University, Beijing, China; ^2^Department of Otorhinolaryngology, Head and Neck Surgery, First Affiliated Hospital of Zheng Zhou University, Zhengzhou, China

**Keywords:** vertigo, vestibular function, hearing audiogram, prognosis, sudden sensorial hearing loss

## Abstract

Sudden sensorineural hearing loss (SSNHL) patients with vertigo have a poorer prognosis. However, the factors associated with hearing recovery remain uncertain. This retrospective study was to evaluate the association between hearing characteristics/hearing recovery and the patterns of vestibulocochlear lesions in SSNHL patients with vertigo. Patients were classified into groups according to the patterns of vestibular dysfunction. We not only compared hearing characteristics and prognosis among subgroups but also determined the potential association between vestibular lesion location and hearing recovery. The shapes of the audiogram differed significantly between patients with normal vestibular function and patients with vestibular dysfunction (*p* = 0.022). Patients whose audiogram indicated profound hearing loss were 3.89 times more likely to have vestibular dysfunction than those whose audiogram shape indicated low-frequency hearing loss (95% CI, 1.02–14.86, *p* = 0.047). Patients who had saccule dysfunction were 0.11 times as likely to have hearing recovery than those who had normal saccule function (95% CI, 0.11–0.31, *p* = 0.001). When adjusted for sex and age, patients who had saccule dysfunction were 0.07 times as likely to have hearing recovery than those who had normal saccule function (95% CI, 0.02–0.22, *p* = 0.001). Abnormal results following cVEMP testing may be a potential predictive factor for poor hearing recovery.

## Introduction

Sudden sensorineural hearing loss (SSNHL) is a disease that is characterized by rapid-onset sensorineural hearing loss of more than 30 dB in at least 3 contiguous audiometric frequencies within 72 h (1). The incidence of SSNHL is approximately 3–27 per 100,000 persons annually, with approximately 66000 new cases per year in the United States (2). The vast majority of cases are unilateral and have additional symptoms, such as tinnitus, vertigo and aural fullness (3, 4). At present, the pathogenesis, clinical manifestations, optimal treatments and prognostic factors of SSNHL remain unclear, with spontaneous recovery rates ranging from 32 to 70% (1, 5).

Due to the close anatomical and phylogenetic association between the cochlea and the vestibular organs, impairment of cochlear function could cause not only SSNHL, but also vestibular disturbance (6, 7). Approximately 30–60% of SSNHL patients also complain about symptom of vertigo, which may appear at the onset of hearing loss or be delayed for hours or even days (1, 8, 9). The presence of vertigo at the time of onset of SSNHL is often regarded as a poor prognosis for hearing recovery (10). However, some SSNHL patients presented vertigo but had mild or moderate hearing loss and a better prognosis. This might suggest the symptom of vertigo was not an independent and determining factor for reflecting the poor prognosis. Recently, some researchers have also found hearing recovery did not differ significantly between SSNHL patients with and without vertigo (11, 12). Vertigo is a symptom of vestibular dysfunction and has been described as a sensation of motion, most commonly rotational motion (13). Any site of vestibular organ disturbance could cause vertigo. Different pathogeneses of SSNHL have been suggested, such as vascular, infectious, oxidative, immunomediated, degenerative, and rupture of the basilar membrane or Reissner's membrane (14–18). Vascular dysfunction was postulated as an important cause of SSNHL because atherosclerotic vascular risk factors and anterior inferior cerebellar artery (AICA) occlusion were tightly associated with SSNHL (19). Different vestibular organs shared different arteries supply with cochlea. Thus, it was reasonable to believe that different patterns of vestibular dysfunction might have different hearing characteristics and prognosis. Research about this was lacking.

Recently, some studies have used vestibular function testing to determine the etiology of SSNHL, such as the caloric test, cervical vestibular-evoked myogenic potential (cVEMP), and ocular vestibular-evoked myogenic potential (oVEMP) (10, 20). The caloric test can be used for exploring lateral semicircular canal function and superior vestibular nerve integrity (21). cVEMP is a method for clinically investigating saccular function and the inferior vestibular pathway, and oVEMP can be used for assessing utricular function and the superior vestibular nerve pathway (22, 23). The combined use of the caloric test, cVEMP and oVEMP has been considered a precise and comprehensive method for locating damaged vestibular regions. A recent study with limited samples indicated extents of profound hearing loss can differ in SSNHL according to patterns of vestibular dysfunction (10). Since severity was tightly associated with prognosis. Thus, we assumed that evaluating the patterns of vestibular dysfunction involved in SSNHL might be useful for the prognostic prediction of hearing loss. To address this issue, we conducted a retrospective study to evaluate the association between hearing characteristics/prognosis and the patterns of vestibular cochlear lesions in SSNHL patients with vertigo.

## Materials and Methods

### Study Design

This retrospective study was approved by the regional ethical standards committee in the First Affiliated Hospital of Zhengzhou University. According to Siegel' criteria of hearing recovery and guideline for sudden hearing loss issued by Chinese Society of Otorhinolaryngology Head and Neck Surgery in 2015 (24, 25), a hearing recovery was defined as an overall magnitude of hearing improvement of at least 15 dB HL; lower levels of improvement were defined as ineffective. We used a test protocol that encompassed the vestibular end organs. Caloric testing was used to assess the lateral semicircular canal. cVEMPs to air-conducted (AC) sound and oVEMPs to bone-conducted (BC) taps were, respectively, used to assess saccular and utricular function. According to Fujimoto's study, we classified patients into different types based on the patterns of vestibular dysfunction (10). All patients presented cochlear damage and were marked C (cochlear) type. Among the C type patients, if a patient presented abnormal cVEMP, oVEMP or caloric responses, we added an S (saccule), U (utricle) or L (lateral semicircular canal). For example, if a patient showed abnormal cVEMPs and oVEMPs but normal caloric responses, we classified the patient as a CSU type. This study consisted of two parts. First, we compared the clinical characteristics, hearing characteristics and hearing recovery between patients with normal vestibular function and patients with vestibular dysfunction and explored the potential hearing factors associated with the presence of vestibular dysfunction. Second, based on the patterns of vestibular dysfunction, the patients were divided into C type, CL type, CU type, CS type, CUL type, CUS type, and CUSL type, and we compared the hearing characteristics and recovery among the subgroups. Finally, we used a logistic regression analysis with two models to investigate the association between the patterns of vestibular dysfunction and hearing recovery.

### Patients

We reviewed the clinical records of new, consecutive patients with SSNHL with vertigo visiting the otolaryngological department at the First Affiliated Hospital of Zhengzhou University between January 2016 and December 2018. All included patients went to see a otolaryngologist and received treatment in the otolaryngology inpatient clinic. All patients had detailed history taken and underwent a series of tests, including physical examination, neurotological examination, imaging, hearing, and vestibular testing. Eye movements were observed by means of an infrared charge-coupled device camera and recorded by electronystagmography (ICS CHARTR 200 VEG/ENG, GN Otometrics). Hearing and vestibular tests were performed within 10 days after onset of symptoms. All studied patients received the same treatment plan which was recommended by Chinese Society of Otorhinolaryngology Head and Neck Surgery (24). The detailed treatment plan was as followings: batroxobin (10 units) in 250 mL of solution intravenously every other day, ginaton (gingko biloba extract) (87.5 mg) in 250 mL of solution intravenously every day, and methylprednisolone (40 mg) in 100 mL of solution intravenously every day for 7 days. After treatment and at the 1-month follow-up, they all also underwent a hearing examination. The diagnostic criteria for SSNHL with vertigo included a sensorineural hearing loss of more than 30 dB, occurring in at least 3 contiguous frequencies in <3 days, and a single attack of vertigo occurring almost simultaneously with the onset of hearing loss. Major exclusion criteria included missing hearing or vestibular data, a previous history of SSNHL in either ear, history of fluctuating hearing and vertigo, history of subjective vertigo episodes, Ménière's disease, migraine, history of ear surgery, history of otosclerosis, congenital hearing loss, physical trauma or barotrauma to the ear, history of genetic hearing loss with strong family history, or craniofacial or temporal bone malformations and central neuropathy.

### Caloric Testing

Caloric nystagmus was recorded in a darkened room by using electronystagmography (ICS CHARTR 200 VEG/ENG, GN Otometrics). Patients lay in the supine position and kept the lateral semicircular canal in a vertical site. We, respectively, used a constant flow of air at alternating temperatures of 30 and 44°C to irrigate the external auditory canal for 30 s. We used maximal slow phase eye velocity for calculating Canal paresis (CP). We defined an abnormal caloric response by the following criteria: CP percentage >20% (26, 27).

### cVEMP Testing

We used surface electrode standing on the upper half of each sternocleidomastoid muscle (SCM), reference electrode placed on the side of the upper sternum and a ground electrode on the chin to record the Electromyographic (EMG) activity. During the recording period, patients stayed in the supine position and were guided to keep their heads raised to shrink the SCM. With the use of Neuropack R, the EMG signal from the stimulated side was amplified and bandpass-filtered (20–2,000 Hz). The stimulation and analysis time were, respectively, 5 Hz and 100 ms. Short tone bursts of 500 Hz (95 dB normal hearing level, 135 dB SPL (peak value), rise/fall time 1 ms, plateau time 2 ms) were also presented. We assessed the latencies and amplitudes of the first positive–negative peaks (p13–n23) of the cVEMP, which were evaluated from the average of two runs. The p13–n23 amplitude on the unaffected side was regarded as Au, and that on the affected side was as Aa. With regard to the assessment of amplitude, the asymmetry ratio for the p13–n23 amplitude (cVEMP AR) was calculated and regarded as 100 [(Au–Aa)/(Aa + Au)]. Based on the data from normal individuals, 34% was the upper limit of the cVEMP AR (28). If reproducible p13–n23 was absent in two runs, we considered it as an “absent response.” If a reproducible p13–n23 appeared and the cVEMP AR (%) was >34%, we considered it as a “decreased response.” Both “decreased response” and “absent response” were considered abnormalities.

### oVEMP Testing

Patients stayed in the supine position and were guided to keep their heads supported by a pillow. We placed surface EMG electrodes on the skin 1 cm below (active) and 3 cm below (indifferent) the center of every lower eyelid, and placed ground electrode on the chin. During the testing period, individuals looked up about 30 degrees straight ahead and held their focus on a small dot approximately 1 m from their eyes. The signals were magnified by an amplifier with the bandwidth of 0.5–500 Hz. The unadjusted signals were averaged (*n* = 50) by Neuropack R. A hand-held 4810 Mini-shaker (Bruel and Kjaer, Naerum, Denmark) with a short rod placed perpendicularly on the forehead at the hairline transferred a BCV stimuli with a 4 ms tone bursts of 500 Hz frequency, in which rise/fall time was 1 ms and plateau time was 2 ms. The peak driving voltage was 80 V and peak force was 128 dB re 1 lN. The applied stimuli and analysis time window were, respectively, 3 times per second and 50 ms. The means of two sets of 50 stimuli each were calculated. We conducted the consecutive runs to affirm the reproducibility of the oVEMP responses. The first negative peak (nI) latency, the subsequent positive peak (pI) latency, and the amplitude between nI and pI were determined from the means of two runs. The nI–pI amplitude on the unaffected side was defined as Au, and that on the affected side was as Aa. With regard to the assessment of amplitude, the asymmetry ratio for nI–pI amplitude (oVEMP AR) was calculated and regarded as 100 [(Au–Aa)/(Aa + Au)]. We used responses recorded from the eye contralateral to the stimulation site to determine the oVEMP AR. Based on the results from normal individuals, 27.3 for the oVEMPs to BCV was the upper limit of normal oVEMP AR (29). If reproducible nI–pI was absent in two runs, we considered it as an “absent response.” If a reproducible nI–pI appear and the cVEMP AR (%) was >27.3, we considered it as a “decreased response.” Both “decreased response” and “absent response” were considered abnormalities.

### Pure Tone Audiogram

We categorized the pure tone audiogram as four types, such as high- or low-frequency hearing loss, flat-type hearing loss, or profound hearing loss. Patients whose average hearing loss at 4–8 kHz was 30 dB larger than that at 0.25–0.5 kHz was defined as high-frequency hearing loss group. Patients whose average hearing loss at 0.25–0.5 kHz was 30 dB larger than that at 4–8 kHz was defined as low-frequency hearing loss group. Patients whose difference between the worst and best hearing levels was <20 dB among six frequencies of 0.25, 0.5, 1, 2, 4, and 8 kHz was defined as flat-type hearing loss group. In the profound hearing loss group, at least 2 frequencies indicated scale-off, and the hearing level was <10 dB than the maximum sound level produced by the audiometer at all six frequencies. The pure tone average (PTA) was calculated with the thresholds at 0.25, 0.5, 1, 2, and 4 kHz.

### Statistical Analysis

SPSS software (version 21.0, SPSS) was used for statistical analyses. For categorical data, frequencies and percentages were calculated to describe the distributions of subgroups among patients according to sex, tinnitus, aural fullness, location of damaged ear, history of diabetes or hypertension, characteristics of the audiogram, duration of vertigo, presence of positional nystagmus, and prognosis effect (recovery). Categorical data were compared with a chi-squared test or Fisher's exact test if appropriate. Means (± SD) were used to summarize the average levels of quantitative data, such as age, time between onset of hearing loss and treatment, and hearing threshold. The Shapiro-Wilk test was used to examine whether the measured values could be approximated by a normal distribution. Quantitative data that were distributed normally were compared by two-sample *t*-test or ANOVA; those that were distributed non-normally were compared by the Wilcoxon signed-rank test. A logistic regression analysis was conducted, and odds ratios (ORs) were calculated for hearing threshold and type of audiogram variables to determine which variables predicted vestibular dysfunction. A multivariable logistic regression analysis, in which age, sex, and patterns of vestibular dysfunction, such as saccule, utricle and lateral semicircular canal, were included as covariates, was used to assess the association between these independent factors and the prognostic effect in SSNHL patients with vertigo. The binary outcome for the logistic regression referred to prognostic effect (effective or ineffective). The 15 dB HL improvement in the PTA was defined as effective. The logistic regression analysis was performed with two models, an unadjusted analysis model and an adjusted analysis model that was adjusted for age and sex. ORs and 95% confidence intervals were calculated according to model-variable coefficients and standard errors, respectively. All *p*-values of <0.05 were considered to indicate statistical significance.

## Results

### Hearing Characteristics and Prognosis of SSNHL Patients With Vertigo

**Figure 1:**
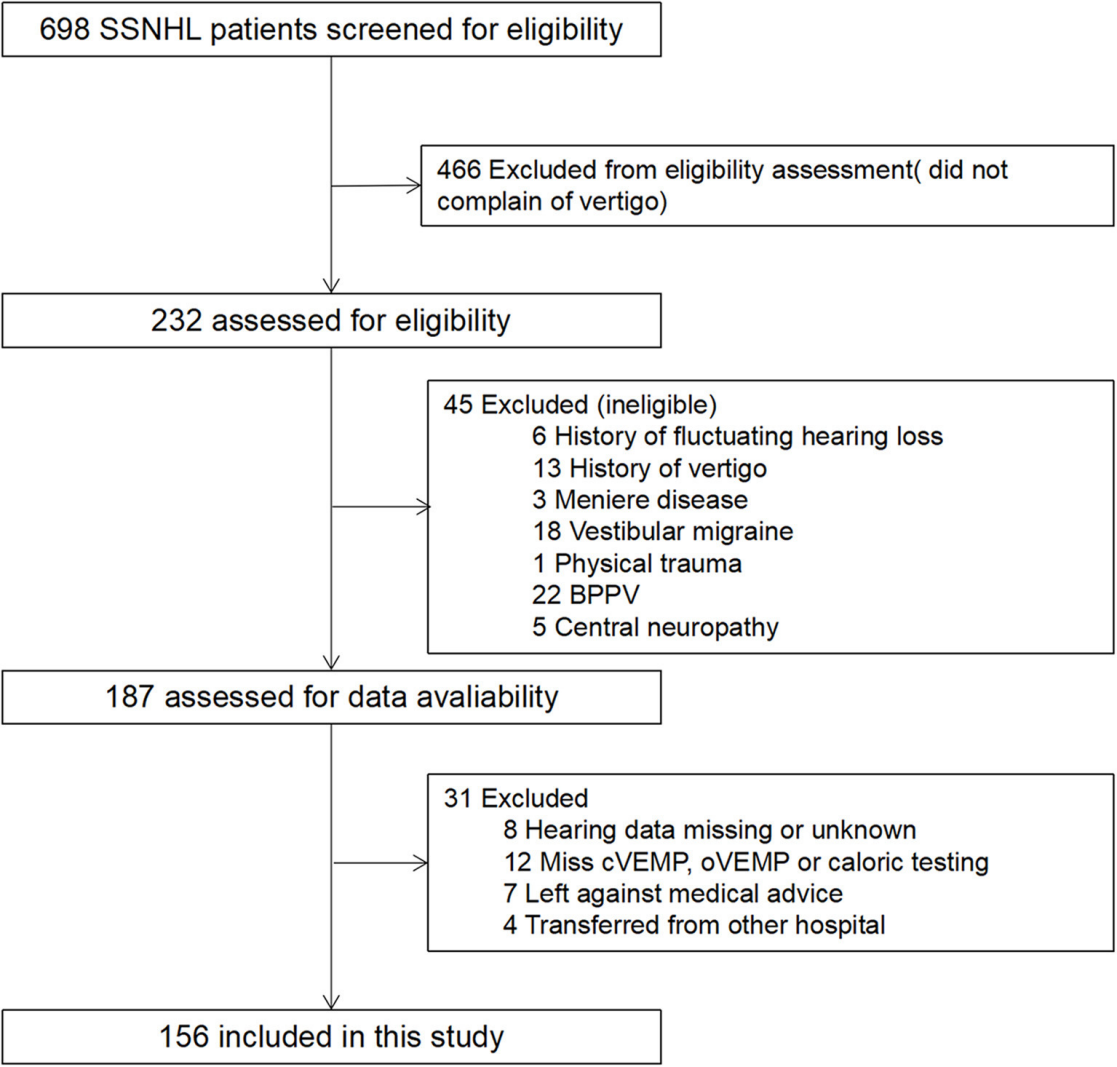
Flow diagram showing study patients screening, eligibility, and inclusion.

During the study period, 232 SSNHL patients who also complained of vertigo were included. Among these patients, 156 patients met the inclusion criteria, and 76 were excluded according to the exclusion criteria (detailed data in Figure 1). The mean (SD) age of the patients was 43.28 ± 14.35 years, and 54.5% were women. Tinnitus and aural fullness were present in 95.5% patients and 69.9% patients, respectively. The average hearing threshold of the affected ear was 67.60 ± 26.92 dB. The mean timing of initial and post-treatment audiogram were 2.53 ± 1.97 and 31.54 ± 3.35 days, respectively. After analyzing the audiogram, 7.1% indicated low-frequency hearing loss, 16.7% high-frequency hearing loss, 32.7% flat-type hearing loss, and 43.6% profound hearing loss. All patients underwent vestibular tests, including caloric testing, cVEMP and oVEMP; 74.4% of patients presented vestibular dysfunction, 60.9% of patients showed abnormal caloric testing, 66.0% of patients showed abnormal oVEMP, and 39.7% of patients showed abnormal cVEMP.

Further analysis between patients with normal vestibular function and patients with vestibular dysfunction was shown in Table 1. Age, sex, PTA s of the affected ear and unaffected ear, duration of vertigo, presence of tinnitus, aural fullness and positional nystagmus, and recovery of hearing did not differ significantly between patients with vestibular dysfunction and patients with normal vestibular function. However, the audiogram shapes of flat type and profound hearing loss differed significantly (*p* = 0.006 and *p* = 0.001) (Table 1).

**Table 1:** Baseline. hearing and prognosis characteristics of SSNHL patients with vertigo.

**Characteristics**	**All studied patients (*n* = 156)**	**Patients with normal vestibular function (*n* = 40)**	**Patients with vestibular dysfunction (*n* = 116)**	***P*-value**
Age, mean, year	43.28 ± 14.35	41.58 ± 14.14	43.87 ± 14.44	0.385
Women, No. (%)	85 (54.5)	20 (50.0)	65 (56.0)	0.509
Left affected ear	82 (52.6)	26 (65.0)	56 (48.3)	0.068
Pure tone average, mean, dB				
Affected ear	67.60 ± 26.92	65.94 ± 27.42	68.18 ± 26.84	0.652
Unaffected ear	27.09 ± 17.19	28.98 ± 20.35	26.44 ± 16.00	0.422
Timing of initial audiogram, mean, day	2.53 ± 1.97	2.75 ± 2.36	2.45 ± 1.82	0.406
Shapes of audiogram, No. (%)				
Low-tone hearing loss	11 (7.0)	5 (12.5)	6 (5.2)	0.119
High-tone hearing loss	26 (16.7)	4 (10.0)	22 (19.0)	0.190
Flat type	51 (32.7)	19 (47.5)	32 (27.6)	0.001
Profound loss	68 (43.6)	12 (30.0)	56 (48.3)	0.006
Other aural symptom, No. (%)				
Aural fullness	109 (69.9)	26 (65.0)	83 (71.6)	0.436
Tinnitus	149 (95.5)	38 95.0)	111 (95.7)	0.856
Duration of vertigo No. (%)				
<1 day	27 (17.3)	8 (20.0)	19 (16.4)	
1–7 days	96 (61.5)	26 (65.0)	70 (60.3)	0.524
>7 day	33 (21.2)	6 (15.0)	27 (23.3)	
Positional nystagmus No. (%)	53 (34.0)	13 (32.5)	40 (34.5)	0.819
Hypertension, No. (%)	28 (17.9)	9 (22.5)	19 (16.4)	0.384
Diabetes, No. (%)	14 (9.0)	5 (12.5)	9 (7.8)	0.353
Timing of post-treatment audiogram, mean, day	31.54 ± 3.35	31.21 ± 3.05	31.65 ± 3.45	0.478
Prognosis, No. (%)				
Recovery	46 (29.5)	16 (40.0)	30 (19.2)	0.091

*cVEMP, cervical vestibular evoked myogenic potentials; oVEMP, ocular cervical vestibular evoked myogenic potentials*.

### Association Between Hearing Characteristics and the Abnormal Vestibular Tests

Logistic regression analysis was used to assess the potential association between hearing characteristics and the presence of vestibular dysfunction in SSNHL patients with vertigo. Patients whose audiogram indicated profound hearing loss were 3.89 times more likely to have vestibular dysfunction than those whose audiogram shape indicated low-frequency hearing loss (95% CI, 1.02–14.86, *p* = 0.047). Age, sex, initial PTA of the affected ear, aural fullness and tinnitus were not significantly associated with the presence or absence of vestibular dysfunction (Table 2).

**Table 2:** Association between hearing characteristics and the abnormal vestibular tests.

**Variables**	**OR (95% CI)**	***P*-value**
Increasing age-per year	1.01 (0.99, 1.04)	0.383
Women	1.28 (0.62, 2.62)	0.509
Increasing initial pure tone average-per dB	1.00 (0.99, 1.02)	0.649
Shapes of audiogram		
Low-tone hearing loss	1	
High-tone hearing loss	4.58 (0.93, 22.59)	0.061
Flat type	1.40 (0.38, 5.23)	0.614
Profound loss	3.89 (1.02, 14.86)	0.047
Aural fullness	1.35 (0.63, 2.91)	0.437
Tinnitus	1.17 (0.22, 6.27)	0.856

### Hearing and Prognosis Characteristics of Subgroups According to Patterns of Vestibular Dysfunction

According to the patterns of vestibular dysfunction, patients were divided into different subtypes. A total of 24.4% were C, 7.1% were CL, 2.6% were CS, 5.1% were CU, 23.7% were CUL, 7.7% were CUS, and 29.5% were CUSL type. Clinical characteristics, hearing characteristics and prognosis were analyzed in these groups (Table 3). Among the vestibular subgroups, age, sex, PTA of the affected ear, symptoms of aural fullness and tinnitus did not differ significantly, but the shapes of the audiogram differed significantly (*p* = 0.002). After treatment for 2 weeks, hearing recovery differed significantly in these subgroups (*p* = 0.001). The percentages of recovery in the C type, CL type, CS type, CU

**Table 3:** Hearing and prognosis characteristics of subgroups according to patterns of vestibular dysfunction.

**Characteristics**	**C group (*n* = 38)**	**CL group (*n* = 11)**	**CS group (*n* = 4)**	**CU group (*n* = 8)**	**CUL Group (*n* = 37)**	**CUS group (*n* = 12)**	**CUSL Group (*n* = 46)**	***P*-value**
Age, mean, year	42.40 ± 14.03	36.27 ± 15.39	43.50 ± 11.56	37.63 ± 16.11	47.78 ± 12.38	35.67 ± 13.90	45.02 ± 14.91	0.069
Women, No. (%)	18 (47.4)	9 (81.8)	1 (25.0)	5 (62.5)	16 (43.2)	10 (83.3)	26 (56.5)	0.069
Left affected ear, No. (%)	25 (65.8)	5 (45.5)	0 (0.0)	5 (62.5)	17 (45.9)	9 (75.0)	21 (45.7)	0.072
Pure tone average, mean, dB								
Affected ear	68.66 ± 25.30	58.41 ± 34.56	56.88 ± 22.53	58.96 ± 35.33	65.67 ± 25.53	77.15 ± 22.51	70.42 ± 27.39	0.626
Shapes of audiogram, No. (%)								
Low-tone hearing loss	3 (7.9)	4 (36.4)	0 (0.0)	1 (12.5)	2 (5.4)	1 (8.3)	0 (0.0)	
High-tone hearing loss	4 (10.5)	1 (9.1)	3 (75.0)	1 (12.5)	6 (16.2)	2 (16.7)	9 (19.6)	0.002
Flat type	19 (50.0)	1 (9.1)	0 (0.0)	2 (25.0)	15 (40.5)	3 (25.0)	11 (23.9)	
Profound loss	12 (31.6)	5 (45.5)	1 (25.0)	4 (50.0)	14 (37.8)	6 (50.0)	26 (56.2)	
Other aural symptom, No. (%)								
Aural fullness	26 (68.4)	8 (72.7)	3 (75.0)	4 (50.0)	29 (78.4)	9 (75.0)	30 (65.2)	0.740
Tinnitus	36 (94.7)	11 (100.0)	4 (100.0)	7 (87.5)	33 (89.2)	12 (100.0)	46 (100.0)	0.228
Duration of vertigo No. (%)								
<1 day	7 (18.4)	1 (9.1)	0 (0.0)	1 (12.5)	4 (10.8)	2 (16.7)	12 (26.1)	
1–7 days	26 (68.4)	4 (36.4)	4 (100.0)	6 (75.0)	22 (59.5)	7 (58.3)	27 (58.7)	0.144
>7 day	5 (13.2)	6 (54.5)	0 (0.0)	1 (12.5)	11 (29.7)	3 (25.0)	7 (15.2)	
Positional nystagmus No. (%)	12 (31.6)	3 (27.3)	4 (100)	3 (37.6)	11 (29.7)	3 (25.0)	17 (37.0)	0.171
Prognosis, No. (%)								
Recovery	14 (26.8)	7 (63.6)	0 (0.0)	4 (50.0)	16 (43.2)	1 (8.3)	4 (8.7)	0.001

type, CUL type, CUS type, and CUSL type patients were 26.8, 63.3, 0.00, 50.0, 43.2, 8.3, and 8.7%, respectively (Table 3).

### Association Between Locations of Vestibular Dysfunction and Prognosis

In this study, we used a logistic regression analysis to explore the association between the locations of vestibular dysfunction and hearing recovery. Two models were used for this analysis. Model 1 was not adjusted for sex and age, and model 2 was adjusted for sex and age. In model 1, younger patients seemed to be more likely to recover hearing (OR, 0.96; 95% CI, 0.93–0.98 *p* = 0.001). Patients who had saccule dysfunction were 0.11 times as likely to recover hearing than those who had normal saccule function (95% CI, 0.11–0.31, *p* = 0.001). Patients who had utricle dysfunction were 0.49 times as likely to recover hearing than those who had normal utricle function (95% CI, 0.24–1.00, *p* = 0.048). Sex and lateral semicircular canal dysfunction were not significantly associated with hearing recovery. In model 2, patients who had saccule dysfunction were 0.07 times as likely to recover hearing than those who had normal saccule function (95% CI, 0.02–0.22, *p* = 0.001). However, utricle dysfunction and lateral semicircular canal dysfunction were not significantly associated with hearing recovery (Table 4).

**Table 4:** Association between locations of vestibular dysfunction and prognosis.

	**Patients, No. (%)**		**Model 1**		**Model 2**	
**Variable**	**Ineffective group (*n* = 110)**	**Effective group (*n* = 46)**	**OR (95% CI)**	***P*-value**	**OR (95% CI)**	***P*-value**
Age, mean, year	45.98 ± 12.99	36.83 ± 15.51	0.96 (0.93, 0.98)	0.001		
Women, No. (%)	56 (50.9)	29 (63.0)	1.65 (0.81, 3.33)	0.167		
Vestibular dysfunction location, No. (%)						
LSC	68 (61.8)	27 (58.7)	0.88 (0.44, 1.77)	0.716	0.67 (0.32, 1.40)	0.290
SA	57 (51.8)	5 (10.9)	0.11 (0.04, 0.31)	0.001	0.07 (0.02, 0.22)	0.001
UT	78 (70.9)	25 (54.3)	0.49 (0.24, 1.00)	0.048	0.52 (0.24, 1.09)	0.084

*LSC, Lateral semicircular canal; SA, Saccule; UT, Utricule*.

## Discussion

In this study, using logistic regression analysis models, we found that vestibular dysfunction was more likely to appear in patients whose audiogram indicated profound hearing loss. In addition, the presence of saccule dysfunction could decrease the possibility of hearing recovery. Our results indicated that saccule function might be a potential factor associated with hearing recovery in SSNHL patients with vertigo.

Many histopathological case studies have been conducted to explore the pathogenesis of SSNHL with vertigo, but their results have been always controversial. Khetarpal et al. (30) reported that vertigo in SSNHL did not result from structural changes in the mechanoreceptors or their nerve but was possibly caused by biochemical changes in the fluids of inner ear. Inagaki et al. (31) and Yoon et al. (32) found that atrophy of the vestibular organs was most frequently present in SSNHL patients. In this study, we used vestibular function tests and found that approximately 74.4% of SSNHL patients with vertigo had vestibular dysfunction while the remaining 25.6% did not. This may suggest that the symptom of vertigo appearing in SSNHL may not actually reflect the presence of vestibular dysfunction.

In this study, we compared the hearing characteristics and prognosis between patients with vestibular dysfunction and patients with normal vestibular function and found that they did not differ significantly in terms of hearing recovery. This might suggest that vestibular function might not be a predictive factor associated with hearing recovery. Our study also suggested that the initial hearing level did not differ significantly between patients with normal vestibular function and patients with vestibular dysfunction, which was consistent with previous studies. Ogawa et al. (33) investigated the association between cVEMP and grade of hearing and found no significant association between initial hearing and cVEMP in 57 SSNHL patients. Nagai et al. (34) discovered that it could not differ significantly between oVEMP and the severity of initial hearing. However, the shapes of the audiogram differed significantly between patients with vestibular dysfunction and patients with normal vestibular function in this study. Subsequently, we used a logistic regression analysis to investigate the potential association between the shapes of the audiogram and the presence of vestibular dysfunction. We found that audiogram indicative of profound hearing loss were potential factors for predicting vestibular dysfunction. Korres et al. (6) also reported a significant association between abnormal VEMP tests and profound hearing loss in 104 SSNHL patients. Thus, in our view, predicting abnormal vestibular function should be based on the shapes of the audiogram, particularly for profound hearing loss, rather than the PTA of the affected ear.

Caloric testing, cVEMP and oVEMP have been used for assessing peripheral vestibular function. Caloric testing was used to clinically assess the lateral semicircular canal; cVEMP and VEMP were used to reflect the function of the saccule and utricle, respectively (10). In this study, we classified the patients into different subgroups according to the patterns of vestibular dysfunction and further analyzed the hearing characteristics to explore their association with patterns of vestibular dysfunction. More cases of vestibular dysfunction appeared in the lateral semicircular canal and utricle than in the saccule. This result was inconsistent with Fujimoto's study, which indicated atrophy of the saccular macula was most frequently present in the vestibular organs of SSNHL patients. They attributed this phenomenon to the anatomy of the saccule in its proximity to the cochlea (10). Our results can be explained by anatomy and pathogenesis. The superior vestibular nerve characterized by longer and narrowed bony canal showed more susceptible to possible ischemic labyrinthine changes or other entrapments when compared with the inferior vestibular nerve or singular nerves (35, 36). In addition, vascular insults have been regarded as one major cause of SSNHL (4). Kim et al. (37) reported that the cochlea and the cristae of the horizontal semicircular canals and utricle developed degenerative changes while the posterior canal ampulla and saccular macula were relatively preserved and proposed that a partial sparing of the inferior vestibular labyrinth might indicate a decreased vulnerability to ischemia due to its better collateral blood supply.

In the present study, 56.2% of CUSL-type patients showed profound hearing loss. Fujimoto et al. conducted a study with a limited number of patients, indicating that profound hearing loss was present in 38% of CUSL patients (10). This might mean that the more extensive the vestibular lesion was, the more severe the hearing loss might be.

Vertigo is a symptom of vestibular dysfunction and has been described as a sensation of motion, most commonly rotational motion (13). A large number of studies have investigated the role of vertigo in SSNHL patients for predicting prognosis and found that SSNHL patients complaining of vertigo might have a poor prognosis for hearing recovery due to their poor hearing (38, 39). However, studies exploring hearing recovery in SSNHL patients with vertigo were rather limited. In the present study, using logistic regression analysis, we found that patients who had saccule dysfunction were less likely to recover than those who had normal saccule function (OR, 0.11; 95% CI, 0.04–0.31; *p* = 0.001; adjusted model, OR, 0.07; 95% CI, 0.02–0.22; *p* = 0.001). This result was consistent with a recent meta-analysis, which reported that the pooled hearing recovery in the abnormal cVEMP response group was nearly half that of the recovery in the normal cVEMP response group (7). Saccules have a better collateral blood supply than other vestibular end organs. When the saccule is damaged, this may suggest that the degree of cochlear damage is severe. The more severe the cochlear damage is, the poorer the hearing recovery may be. This is in contrast with Niu's study, which suggests that lateral semicircular canal function may be related to the severity of cochlear damage due to the more profound hearing loss appearing in patients with an abnormal caloric test (8). Our results indicate that abnormal results from cVEMP testing may be a potential predictor of the impossibility of hearing recovery.

Our study may have several limitations. First, it is difficult to maintain homogeneous sample in each subcategory in this retrospective study, which may have caused selection bias and information bias. Second, vestibular tests were not carried out at SSNHL onset, and some patients were admitted to the hospital days later. Vestibular function might have recovered before the vestibular tests were performed. This may have increased the number of patients with normal vestibular function. Third, we did not assess the functions of the anterior and posterior semicircular canals in this study. Those patients who were classified as C type might have also had dysfunction of the anterior and posterior semicircular canals. In addition, before going to the hospital, the patients did not undergo vestibular tests, and thus we did not know if the results would have been abnormal.

In conclusion, the percentages of abnormal vestibular responses in SSNHL patients with vertigo were highest in caloric testing, followed by oVEMP and cVEMP. Abnormal cVEMP testing may be a potential predictive examination for poor hearing recovery.

## Data Availability Statement

The raw data supporting the conclusions of this article will be made available by the authors, without undue reservation.

## Ethics Statement

The studies involving human participants were reviewed and approved by the regional ethical standards committee in the First Affiliated Hospital of Zhengzhou University. The patients/participants provided their written informed consent to participate in this study.

## Author Contributions

YW built the models, analyzed the data, and drafted the manuscript. LW and FY were involved in reviewing the manuscript. LY was the principal investigator and managed the study. All authors read and approved the final manuscript.

## Conflict of Interest

The authors declare that the research was conducted in the absence of any commercial or financial relationships that could be construed as a potential conflict of interest.
